# Are You HIV Invincible? A Probabilistic Study of Discordant Couples in the Context of HIV Transmission

**DOI:** 10.1371/journal.pone.0094799

**Published:** 2014-05-19

**Authors:** Georgiy Bobashev, Jacob Norton, Wendee Wechsberg, Olga Toussova

**Affiliations:** 1 RTI International, Durham, North Carolina, United States of America; 2 North Carolina State University, Raleigh, North Carolina, United States of America; 3 Pavlov Medical University, St.-Petersburg, Russia; University of Missouri-Kansas City, United States of America

## Abstract

A number of factors have been identified that are related to sexual and injecting HIV transmission. We developed a probabilistic mathematical model to put these factors together and interpret risks in the context of individual behavior among injecting drug-using (IDU) couples in St. Petersburg, Russia. Some HIV-discordant couples have unprotected sex and sometimes inject drugs together but stay discordant for a long time, while some individuals acquire HIV on the first encounter. We considered existing estimates of HIV transmission risks through injecting and sexual contacts to develop a predictive survival model for an *individual* who is exposed to HIV through intimate relationships. We computed simulated survival curves for a number of behavioral scenarios and discussed sources of simulated uncertainty. We then applied the model to a longitudinal study of HIV-discordant couples and validated the model’s forecast. Although individual prediction of seroconversion time appeared impossible, the ability to rank behavioral patterns in terms of HIV risk and to estimate the probability of survival HIV-free will be important to educators and counselors.

## Introduction

An HIV-negative woman has been having unprotected sex with her HIV-positive partner for several months and remains HIV free. This gives her a strong belief that she cannot get infected with HIV and thus is “invincible” (i.e., does not need to use protection or care about getting the disease). In this study we examine probabilistically the chances of staying HIV free just by luck, and how different behaviors change these chances. Having such an argument substantiated would help public health and harm reduction counselors to convince people at risk to change their risky behavior patterns.

Interpretations of HIV risks can be confusing. On one hand, around 50,000 new HIV infections occur annually in the United States [1]. On the other hand, the estimated per act transmission probabilities remain relatively small (e.g., probability of female-to-male transmission during unprotected vaginal intercourse is about 0.004–0.005) [2], [3], which might send the wrong message about the danger of unprotected sex, thus leading to a dichotomy of beliefs and perceptions. At one extreme there is an elevated public scare against HIV to the point that some people are afraid to shake hands with an HIV-positive person. At the same time, there are high-risk individuals who have had sexual and injecting encounters with infected individuals and would do it again regardless of HIV status. Additionally, a perception of “invincibility” based on staying HIV negative could lead to behaviors with higher transmission rates. The link between seemingly small per act transmission probabilities and high incidence is in the repetitive risk behavior, which compounds individual per act probabilities. Individual infectivity adds a complicating dimension to HIV transmission. Infectivity strongly depends on an individual’s viral load, which in turn is related to the stage of infection and the use of antiretroviral treatment.

Generic recommendations to use condoms, reduce the number of sex partners, and not share used syringes and other blood-containing objects would be more compelling if they were supported by numeric examples. So far, little has been done to estimate *individual* probabilities of becoming HIV positive based on individual lifestyle. Providing numeric values of HIV probabilities to at-risk subjects and showing how much changes in lifestyle (e.g., increased condom use) can decrease probabilities of acquiring HIV has implications for behavioral counselors and adds scientific argument to the behavioral change message.

Predictive modeling provides a bridge between theoretical knowledge and practical implications for behavioral change. There is a sizable body of literature that estimates HIV transmission per unprotected vaginal, anal, and oral act [2]–[4] and the effects that condoms have on the reduction of this probability [3], [5]. Additionally, there is literature on HIV transmission through sharing syringes [6], [7], [15] and the effects of viral loads and antiretroviral therapy (ART) medication on disease transmission [8]–[12]. Behavioral surveys of discordant couples provide a description of individual risky activities that could be used to calculate the compounded risks and provide feedback to subjects.

In this study, a model was developed that predicts staying HIV free with an HIV-positive partner. A review of published peer-reviewed HIV transmission rates was used to parameterize the model, and the model was validated using data from a discordant couples study, conducted in St. Petersburg, Russia. First, the conceptual model, its literature-based parameterization, and the way it makes projections are presented. Then, a description of the study and the data used to validate the model and a discussion of the results and their implication for risk reduction counseling are presented.

## Methods

### Ethics Statement

Participation in the study was confidential. All real couple identification numbers (ID) have been masked for this article. The study protocol was reviewed and approved by the Institutional Review Board (IRB) of the Biomedical Center, St. Petersburg, Russia.

### Probabilistic Model

The central piece of our methodology is a probabilistic model that estimates the probability of risk transmission from an HIV-positive to an HIV-negative person after several sexual and jointly injecting acts. Denote a probability of transmission per single act as p then, assuming independence between transmissions from one act to another, after *N* acts the probability will be
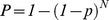
(1)


Considering a variety of sexual and drug use acts, each act can have a different transmission probability. Then, after M sexual and K injecting sharings the total probability becomes
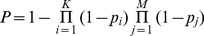
(2)


The remaining challenge is estimating the transition probabilities, which are accomplished by reviewing the literature. For simplicity, the probability of transmission during a single act of vaginal intercourse is considered the reference. This reference probability is modified to represent unprotected anal and oral intercourse and the use of condoms. Transmission probabilities associated with syringe sharing were calculated for episodes where the HIV-negative partner injected after an HIV-positive person. The types of syringes and the effects of syringe cleaning were used as the modifiers. As indicated by studies of syringe use [13], in Russia drug users use high dead-space syringes and thus the risk of HIV transmission is elevated. Viral load was considered an important modifier for all transmission probabilities. Following the logic in Leynart et al. [14], we assumed that during the acute stage transmission is 10 times higher than during the clinical latency (chronic) stage, and if an individual is undergoing ART because of reduction in viral load, the transmission is lower by 75% [8]–[12]. Model parameters and the sources from which they were obtained are presented in Table 1. Assuming a reported or hypothetical schedule of various sexual and joint injecting acts these parameters can be used in equation (2) to generate individual survival for an HIV-negative person, visualized in the form of survival curves. The model can then be validated on longitudinal data given that the sample size and the number of risky acts are large enough to produce positive incidence.

**Table 1:** Model parameters.

Parameter Description	Value	Source
Transmission probability for using a syringe after an infected partner, assuming unsafesyringe use, infected partner in latency stage, and infected partner untreated.	0.007	Chin, 1992; Hudgens et al., 2001; Kaplan & Heimer, 1992; Wilson et al., 2008
Transmission probability for male-female vaginal sex, assuming no condom, infectedpartner in latency stage, and infected partner untreated. This probability is used as areference for sexual transmission probabilities.	0.004	Royce et al., 1997
Relative risk of infection if infected partner is being treated with ART	0.25	Attia et al., 2009; Baggaley et al., 2013; Wilson et al., 2008
Relative risk of infection if condom is used	0.1	Varghese et al., 2002, Weller et al., 2011
Relative risk of infection for vaginal insertive sex	2	Varghese et al., 2002
Relative risk of infection for anal insertive sex	1.3	Varghese et al., 2002
Relative risk of infection for anal receptive sex	5	Varghese et al., 2002
Relative risk of infection for oral sex	0.1	Varghese et al., 2002

Obtained from peer-reviewed literature and educated guesses.

### Study Population

A pilot longitudinal study that was conducted with serodiscordant couples in St. Petersburg, Russia, in 2009–2011 was considered for model validation. The goal of the study was to conduct a needs assessment to understand behavioral risks and how to best implement risk reduction interventions for serodiscordant couples. The couples were recruited from ongoing projects at the Biomedical Center and at the Municipal AIDS Center of St. Petersburg. *Discordant couple* for this study was defined as a couple with different HIV statuses who practiced unsafe sex behavior before or after the HIV-positive status of one of the partners had been revealed. One or both of them could be injecting drug users. The couples were separately assessed at baseline and then 6 months after enrollment. The couples were interviewed separately about their sexual and drug-injecting behavior and underwent HIV counseling and testing. Participation in the study was confidential. All real couple identification numbers (ID) have been masked for this article. The study protocol was reviewed and approved by the Institutional Review Board (IRB) of the Biomedical Center, St. Petersburg, Russia. Participants received condoms or phone or gift cards for participation in the study.

#### Assessments

Participants completed face-to-face interviews that took around 30 minutes. The interview consisted of sociodemographic questions; sections on alcohol and drug use, treatment experience, health evaluation, and knowledge about HIV/AIDS; and detailed questions about sexual and injecting behavior. Items came from the Survey used in the cohort studies in St. Petersburg. A special section for relationships with partners was developed for this study. A codebook for the variables used in our model is presented in Table S1. After HIV counseling, participants underwent HIV-1 testing by enzyme immunoassay with confirmatory Western blot analysis. For HIV-positive participants viral load was assessed.

#### Demographic and behavioral characteristics of the sample at baseline

Twenty-nine heterosexual couples participated in the study. Thus, in the sample of 58 people there were 29 HIV-positive people (18 males and 11 females) and 29 HIV-negative people (11 males and 18 females). The average age was 31, ranging from 20 to 50 years old. Most participants (88%) were either legally married or in a civil marriage; 88% of the sample had completed secondary or higher levels of education, 36% of the sample was unemployed, those 64% of the sample who were employed had average and high legal income in the past 3 months. A total of 83% of the sample lived in their own apartments and there were no homeless people in the sample.

Seven HIV-negative and 13 HIV-positive people (35%) did not use alcohol in the past 3 months, 21 HIV-negative and 28 HIV-positive people (85%) injected drugs in the past but only 6 and 5, respectively (18%), were current injectors. A total of 65% of the sample did not use condoms with their main partner. One-third of couples had been in a relationship for less than 1 year, another third was in a relationship between 1 and 2 years, and the last third were in a relationship longer than 2 years. A total of 26 HIV-positive partners (92%) were registered at the Municipal AIDS Center and 7 people (27%) were using ART.

Four couples either split up or were lost to follow-up before the end of the 6-month study. None of the subjects were in the acute phase of HIV and none were in the AIDS phase, so we assume that all were in the stage of chronic HIV infection (clinical latency).

## Results

### Theoretical Model

**Figure 1:**
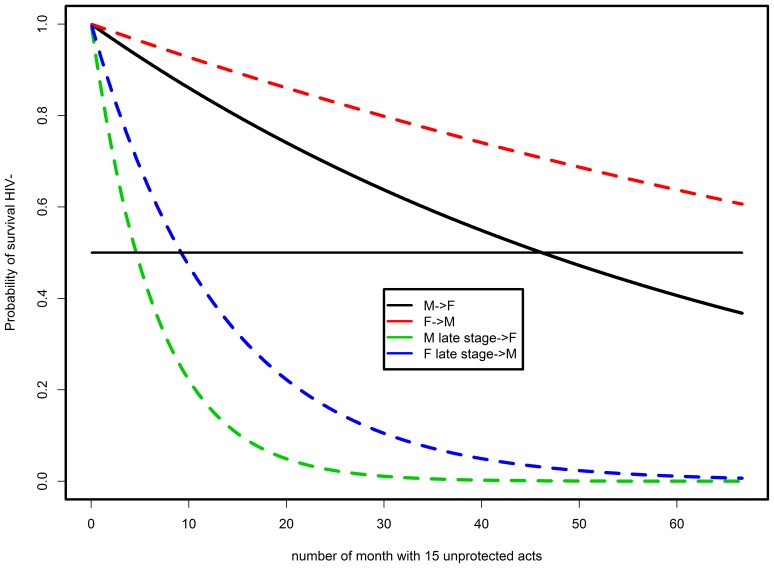
Examples of theoretical curves for survival without HIV under the assumption that the sex partner is HIV positive, and assuming two sexual intercourses per week.

Figure 1 presents survival curves for individuals who practice unsafe sex twice a week separately for male-to-female transmission and female-to-male transmission. Viral load was considered in two disease stages: for chronic and AIDS stages. We also include a solid horizontal reference line that indicates a 50% chance of becoming infected. The model shows that under these assumptions it takes more than 3 years (about 45 months) for half of male HIV-positive and female HIV-negative couples to convert. Thus, it is not surprising that a woman having regular sex for several months would not get infected. When the viral load is increased, such as in the AIDS stage, the probability shifts dramatically with only 6 months as the expected time when half of the couples convert. Similar arguments could be made for the early (acute) stage of infection.

### Validation on Real Data

The model was applied to the real data, and for each subject in the study probability of conversion was calculated. Although the model is probabilistic and is not intended to correctly tell who will and will not seroconvert at 6 months, individuals who were not lost to follow-up were ranked according to highest risk. Two HIV-negative partners (1150 and 2423) seroconverted. Couple 2956 was already converted by the time of the baseline HIV test. ART was used by the HIV-positive individual in couples 3210, 3612, 7015, 5419, 2717, 3330, and 7532.

Both answers from HIV-positive and HIV-negative subjects were considered. A general tendency in the reported risk was that HIV-positive people reported either the same or less risky behavior than the HIV-negative partner. Only in four couples did an HIV-positive person report more risky behavior. Results for the followed couples are presented in Table 2.

**Table 2:** Probability of infection and uncertainty because of potential reporting bias.

Couple ID	Probability of getting infected in 6 months	Difference in 6-month survival reported by HIV-positive and HIV-negative subjects. Negative values mean that the HIV-positive person reported more risky behavior	Sex of HIV-negative person	ART
5633	0.4941	0.101	M	N
**2423**	**0.4704**	**0.089**	**F**	**N**
**1150**	**0.3464**	**0.013**	**M**	**N**
2266	0.3028	0.127	F	N
2717	0.2863	0.043	M	Y
6310	0.1702	−0.219	F	N
2648	0.1043	−0.176	M	N
1403	0.0998	0.169	F	N
7713	0.0873	0.122	F	N
9811	0.0825	0.260	M	N
8670	0.0771	0.012	F	N
2956	0.0770	0.023	M	N
3210	0.0673	0.016	M	Y
2807	0.0639	0.060	M	N
3009	0.0575	0.008	F	N
9607	0.0411	0.019	M	N
5419	0.0395	−0.073	F	Y
7532	0.0265	0.003	F	Y
1817	0.0239	0.045	M	N
7118	0.0119	0.023	F	N
9314	0.0113	0.002	F	N
2192	0.0095	0.010	M	N
3612	0.0089	0.006	F	Y
2368	0.0055	0.005	F	N
3330	0.0044	0.003	F	Y
1631	0.0019	0.008	F	N
7015	0.0000	0.006	F	Y

Two individuals seroconverted at 6 month (marked in bold).

**Figure 2:**
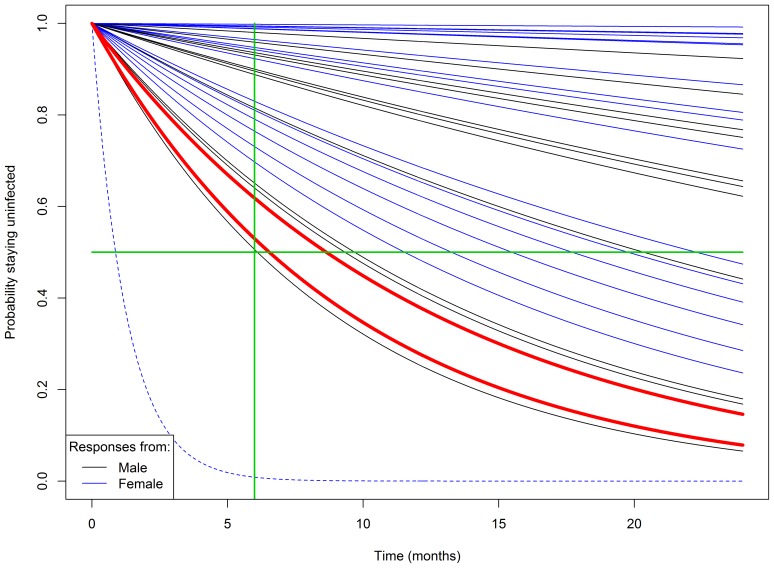
Projected probabilities of remaining HIV negative for study participants. Subject 2445 had the highest probability to seroconvert but got lost to follow-up; thus, her survival curve is represented with a dotted line. Subjects who seroconverted after 6 months are highlighted in red. The vertical line corresponds to 6-month follow-up check and the horizontal line corresponds to a 50% probability of seroconversion.

The two subjects who converted over the 6-month period are not surprisingly on the top of the risk list. Given the estimated probabilities, the expected number of seroconversions in 6 months is 2.8 with a 95% uncertainty interval (0, 6.1). Thus, the result is well within plausible numbers, especially considering that one of the highest risk couples was lost to follow-up. Figure 2 presents a graph projecting survival without seroconversion over time. The main behavioral assumption is that the couples continue their reported activities.

Sensitivity analysis of the results was conducted by perturbing the parameters within a 10% range. Perturbation of the parameter values resulted in variation in the survival estimates ranging from 0 (at time zero) to 9% (at 8 months), which illustrates the nonpropagation of error in the survival model with constant hazard.

## Discussion

As simple as this model may appear, it has shown to forecast individual probabilities of HIV transmission among discordant couples. By incorporating estimates of sexual and injecting risks with individual lifestyles, the model translates generic risks into individual probabilities of infection. The model explains that it is not unusual for a discordant couple to remain discordant for several months or even years.

The model was applied to the survival of the discordant pairs in a cohort study and showed that the model results agree with the observed incidence. The model could be used to illustrate what happens under different “what if” scenarios, such as reduction in risky behavior and effects of ART. Thus, the model provides a tool for harm reduction counselors to illustrate how behavior change could help partners stay healthy and the role of different factors in transmission. The implication of this approach is especially important for those discordant couples who are deliberately not using protection (e.g., if they are trying to conceive a child). The model will provide a way to evaluate the risks associated with sexual activities and emphasize the use of ART and strategies for condom use.

Although the model was applied to discordant couples, it has the possibility to be further expanded to represent more complex relationships including multiple partners and complex sexual/injecting networks. If these networks are not precisely defined, individual predictions become less reliable and lean toward aggregate values because of uncertainty with the HIV status of the intimate partners. Nevertheless, the relative effects of sexual and injecting risks will remain the same.

The model also illustrates the three major sources of heterogeneity in HIV transmission. First, risk can differ by orders of magnitude from one activity to another (e.g., unprotected insertive vaginal and unprotected receptive anal intercourse risks differ by a factor of 10). Second, individual behavior (frequency) contributes almost equally to heterogeneity in individual probabilities and incidence. Third, viral load has been shown to have a dramatic effect on HIV transmission. The use of ART is a critical component in the reduction of risk.

It is imperative to understand the implications of a probabilistic approach. Although the probability of acquiring a disease for some activities can seem low, HIV transmission can occur at any of the acts. Regardless of how long it has been since the start of the intimate relationship, for the same activity (e.g., unprotected receptive anal sex) transmission can occur with the same probability during the first unprotected act and after a year of regular sexual encounters.

The accuracy of the model is limited by the accuracy of the self-reports, uncertainty in the parameter estimates, and the intrinsic uncertainty in making forecasts. Behavior tends to change over time, which shifts probabilities. The bigger uncertainty is, however, the accuracy of parameter estimates, which need to account for the intensity of the activity and a number of other factors. When calculating the risk of HIV transmission by injecting with the others, the model considers background HIV prevalence among injecting drug users. This component arguably carries the most uncertainty in the model. Additional sensitivity analysis (increased and decreased injecting related parameters by 50%) showed no change in the *ordering* of seronegative individuals to seroconvert from most likely to least likely. This result is somewhat expected because only a few individuals injected with “strangers” in our sample, and most seronegative injectors also injected with their HIV-positive partners. However, for other samples and subpopulations it could potentially have a stronger effect.

It is also important to consider that although the model is focused on individual probability, it does not predict the actual timing of the event; however, the mean expected time until seroconversion could theoretically be calculated. Nevertheless, reporting of expected time would be misleading for prevention purposes because it has little to do with the actual seroconversion and can create unnecessary confusion.

In conclusion, the study developed and validated a model that could be used in education, HIV prevention, and counseling of HIV-discordant couples, illustrating the impact of individual behavior on potential health trajectories.

## Supporting Information

Table S1 Codebook for the variables use in the study.
(DOCX)
