# Supplementary material for: Are You HIV Invincible? A Probabilistic Study of Discordant Couples in the Context of HIV Transmission
Source: PLoS One. 2014 May 19;9(5):e94799. doi: 10.1371/journal.pone.0094799 (PMC4026137; doi:10.1371/journal.pone.0094799)
Supplement: Table S1 — Codebook for the variables use in the study. (DOCX) [file pone.0094799.s001.docx]

# Supplementary Materials

Table S1. Codebook for the variables used in the study.

| Variable Name | Variable description, codebook |
| --- | --- |
| ID | ID |
| ID1 | ID for a couple |
| HIV | HIV status (0 = negative) |
| Gender0M | Gender (0=male, 1=female) |
| SexVTotal | Total number of vaginal sex acts past year |
| SexVProt | Total number of vaginal sex acts past year with condom |
| SexVunPr | Total number of vaginal sex acts past year without condom |
| SexATotal | Total number of anal sex acts past year |
| SexAProt | Total number of anal sex acts past year with condom |
| SexAunPr | Total number of anal sex acts past year without condom |
| InjectSharingCat | In the last month how many times you used drugs with other people? 0=never, 1=1-2 times, 2=several times, 3= sometimes, 4= often, 5= always |
| LWUsedSyringeAfterSomeone | In the last week how often you have used a syringe after someone else? 0= never, 1= less than once a week, 2= 1-2 times a week, 3= 3-4 times a week, 4= 5-6 times a week, 5= every day once a day, 6= every day 2-5 times a day, 7= more than 7 times a day |
| LWUsedSyringeAfterHIV | In the last week how often you have used a syringe after someone whom you know is HIV positive? 0= never, 1= less than once a week, 2= 1-2 times a week, 3= 3-4 times a week, 4= 5-6 times a week, 5= every day once a day, 6= every day 2-5 times a day, 7= more than 7 times a day |
| LWSharedWaterCleaning | In the last week how often you have shared syringe cleaning water? 0= never, 1= less than once a week, 2= 1-2 times a week, 3= 3-4 times a week, 4= 5-6 times a week, 5= every day once a day, 6= every day 2-5 times a day, 7= more than 7 times a day |
| LWSharedCup | In the last week how often you have shared equipment (spoon, cup)? 0= never, 1= less than once a week, 2= 1-2 times a week, 3= 3-4 times a week, 4= 5-6 times a week, 5= every day once a day, 6= every day 2-5 times a day, 7= more than 7 times a day |
| LWSHaredCotton | In the last week how often you have shared cotton? 0= never, 1= less than once a week, 2= 1-2 times a week, 3= 3-4 times a week, 4= 5-6 times a week, 5= every day once a day, 6= every day 2-5 times a day, 7= more than 7 times a day |
| LWSharedDrugsyringtosyring | In the last week how often you have used the drug which you transferred from someone else’s syringe? 0= never, 1= less than once a week, 2= 1-2 times a week, 3= 3-4 times a week, 4= 5-6 times a week, 5= every day once a day, 6= every day 2-5 times a day, 7= more than 7 times a day |
| LWUsedBleach | In the last week how often you have cleaned syringe with bleach? 0= never, 1= less than once a week, 2= 1-2 times a week, 3= 3-4 times a week, 4= 5-6 times a week, 5= every day once a day, 6= every day 2-5 times a day, 7= more than 7 times a day |
| LWUsedWater | In the last week how often you have cleaned syringe with water? 0= never, 1= less than once a week, 2= 1-2 times a week, 3= 3-4 times a week, 4= 5-6 times a week, 5= every day once a day, 6= every day 2-5 times a day, 7= more than 7 times a day |
| LMSharedWith | In the last month when you shared injecting equipment did you do it with: 0= always with the same person, 1=mostly with the same person, 2= sometimes with different people but in the same group, 3=more than with one person and from different groups |
| LMHowManyPeople | If more with one person, with how many people? |
| LMTotalPeopleShare | With how many different people did you share the equipment in the past month? |
| LMTotalSyringeUse | On average how many times you use syringe before discarding it? |
